# Inhibition of α-hemolysin activity of *Staphylococcus aureus* by theaflavin 3,3’-digallate

**DOI:** 10.1371/journal.pone.0290904

**Published:** 2023-08-31

**Authors:** Anna Goc, Waldemar Sumera, Matthias Rath, Aleksandra Niedzwiecki

**Affiliations:** Department of Infectious Diseases, Dr. Rath Research Institute, San Jose, California, United States of America; Indiana University School of Medicine-Northwest, UNITED STATES

## Abstract

The ongoing rise in antibiotic resistance, and a waning of the introduction of new antibiotics, has resulted in limited treatment options for bacterial infections, including these caused by methicillin-resistant *Staphylococcus aureus*, leaving the world in a post-antibiotic era. Here, we set out to examine mechanisms by which theaflavin 3,3’-digallate (TF3) might act as an anti-hemolytic compound. In the presented study, we found that TF3 has weak bacteriostatic and bactericidal effects on *Staphylococcus aureus*, and strong inhibitory effect towards the hemolytic activity of its α-hemolysin (Hla) including its production and secretion. A supportive SPR assay reinforced these results and further revealed binding of TF3 to Hla with KD = 4.57×10^−5^ M. Interestingly, TF3 was also able to protect human primary keratinocytes from Hla-induced cell death, being at the same time non-toxic for them. Further analysis of TF3 properties revealed that TF3 blocked Hla-prompting immune reaction by inhibiting production and secretion of IL1β, IL6, and TNFα *in vitro* and *in vivo*, through affecting NFκB activity. Additionally, we observed that TF3 also markedly attenuated *S*. *aureus*-induced barrier disruption, by inhibiting Hla-triggered E-cadherin and ZO-1 impairment. Overall, by blocking activity of Hla, TF3 subsequently subdued the inflammation and protected the epithelial barrier, which is considered as beneficial to relieving skin injury.

## Introduction

*Staphylococcus aureus (S*. *aureus)* is a prevalent, Gram-positive, and non-motile coccus associated with skin and soft tissue infections (SSTI), as well as with sepsis and pneumonia [1–4]. This bacterium is vastly responsible for the infections in healthcare environments and in the communal milieus. In the 1960s the first methicillin-resistant *S*. *aureus* (MRSA) was identified, and it would later become endemic in hospitals. Meanwhile, in the 1990s, several community-associated methicillin-resistant *S*. *aureus* strains (CA-MRSA) emerged, which are now posing a major global challenge [4–8]. Over time it became apparent that *S*. *aureus* had positioned itself as a challenging pathogen and a leading cause of common bacterial infections in humans [9]. The pathogenicity of *S*. *aureus* is associated with numerous virulence factors, including cell surface proteins (e.g., protein A, adhesins), and secreted enzymes and toxins (e.g., hemolysins and leukocidins). These are associated with a variety of toxic effects, including tissue damage, promotion of bacterial dissemination, and evasion of the host innate system. The CA-MRSA strain type USA300 has been acknowledged as more virulent than customary hospital-associated strains, in part owing to relatively higher expression of virulence factors e.g., phenol-soluble modulins (PSMs) and α-hemolysin (α-toxin, Hla), at the same time being highly multidrug resistant in nature [10–18]. Recognizing that *S*. *aureus* easily colonizes the nasal, mucosal, and tissue routes, that it is persistently carried by as many as 30% of healthy adults (which renders more than 100 million people susceptible in the United States alone), and that skin wounds affect more than 6 million individuals in the United States annually, which parallels to annual costs of $25 billion for the healthcare system, *S*. *aureus* has been classified as a “serious threat” by the Centers for Disease Control and Prevention (CDC) [1–9]. *S*. *aureus* infections are initiated through trauma to the skin or mucosal layer, and progress through an invasive or toxin-mediated process. The list of pathogenic disorders includes disruption of the epithelial barrier, inhibition of opsonization, neutrophil cytolysis, interference with neutrophil chemotaxis, and inactivation of antimicrobial peptides [10–18].

Alpha-hemolysin (α-hemolysin, α-toxin, Hla) is a pore-forming protein with cytolytic activity toward a variety of cell types, including human keratinocytes and platelets, epithelial and endothelial cells, monocytes and lymphocytes [11–25]. It functions primarily as an instrument to adapt host tissue to a nutrient source for the bacteria. It is produced by almost all virulent strains of *S*. *aureus* and is a concern in several diseases, including skin and soft-tissue infections (SSTIs) and pneumonia. Seilie and Bubeck Wardenburg demonstrated that Hla is essential for pathogenesis in a mouse model of CA-MRSA pneumonia and activation of NFκB signaling pathway [22]. Bartlett *et al*. showed that Hla prompts severe lung inflammation through forcing production of CXC chemokines by host cells during *S*. *aureus*-induced pneumonia [26]. Kwak *et al*. reported on reduction of protein associated with tight (ZO-1, ZO-3, and occludin) and adherent (E-cadherin and β-catenin) junctions at cellular levels, while Liu *et al*. showed activated MAPK signaling pathway upon Hla stimulation [27, 28]. Hla is secreted as a water-soluble, 34 kDa monomer, isoelectric at pH 8.5 to 8.6, that binds to target cellular membranes and then oligomerizes to form membrane-inserted heptameric pores 10 nm in diameter [18, 19]. The *hla* gene was cloned and sequenced by Kehoe *et al*., who showed that this gene is present in a single copy in the bacterial chromosome [29]. Expression of Hla is regulated, at least in part, by the two”machineries”, the Agr regulatory system, and the SaeR/S signal transduction system, which regulates the production of several exoproteins, including staphylokinase and alpha-, beta-, and delta hemolysins, as well as serine and metalloproteases [30–34]. Its discovery explained several earlier observations, e.g., that production of many exoproteins in *S*. *aureus* is strictly synchronized and appears mainly during the post-exponential or stationary phase. Binding of Hla monomers to the cellular membrane triggers their oligomerization to a 232.4-kDa membrane-inserted heptamer [21, 22]. Hla interacts with specific binding sites on cells (receptors) but can also engage in a non-specific interaction with lipid layers. Both types of binding results in the formation of pore-forming oligomers. Although, this protein does not contain cysteine, its central region is rich in glycine residues, which also have a high probability of random coil, hence its ability to fold between the C- and N-domains. In contrast to other porins (e.g., complement component C9, and *E*. *coli* hemolysin), Hla does not require bivalent cations such as Ca^2+^ for binding and cytolytic function [23]. The work of Bayley *et al*. and Bhakdi *et al*. established an oligomerization model, by which a Hla works, as follows: binding of monomers to the lipid membrane, upon which they oligomerize, forming hexamer that spontaneously insert into the membrane, causing its bridging and penetration, ultimately forming an amphipathic channel across the lipid bilayer with an inner diameter of about 1.5 nm pore [23, 32]. Resulting conformational changes are followed by disruption of the cellular membrane. Examples of subsequent ramifications include: influx of extracellular Ca^2+^ into the cell, causing in turn hydrolysis of membrane phospholipids and metabolism of arachidonic acid to leukotrienes, proteinoids, and thromboxane A2, an activation of the protein kinase C, and the induction of NFκB nuclear translocation, causing pro-inflammatory stimulus production, e.g., IL1β, IL6, TNFα, and IL8 [35–39]. In the epithelial cells, which are key targets of Hla, E-cadherin is a main substrate for ADAM10, which is a receptor for Hla. An activation of ADAM10, a zinc-dependent metalloprotease expressed as a type I transmembrane protein on the surface of most host cells, involves degradation of E-cadherin, which compromises the tissue barrier function, in turn facilitating *S*. *aureus* invasion. All this projects the loss of the characteristic “*milieu intérieur*” required to sustain homeostasis and cell death. The pathogenic relevance of Hla is based on the knowledge that (i) certain human cells, including monocytes, endothelial cells, and platelets, express high-affinity binding sites that are effectively attacked by the toxin under physiological conditions, and (ii) damage occurring to these cells triggers pathological sequelae [39–41].

Theaflavins (TFs) are a class of polyphenolic compounds consisting of a benzotropolone skeleton with or without the galloyl esters residues. They are responsible for the brownish pigment of black tea, accounting for about 2–6% of their dried leaves (*Camellia sinensis*, fam. *Theaceae*). TFs are formed during the oxidation process of selected catechins (epicatechin and epigallocatechin-3-gallate) in the presence of polyphenol oxidase and peroxidase enzymes [42–45]. During fermentation, the catechins get converted to TFs, primarily to theaflavin (TF1), theaflavin-3-gallate (TF2A), theaflavin-3’-gallate (TF2B), theaflavin-3,3’-digallate (TF3), as well as certain polymers of thearubigin [28]. Several health benefits of TFs have been identified, such as: anti-obesity, anticancer, anti-atherosclerotic, anti-inflammatory, anti-viral, anti-bacterial, anti-osteoporotic, and anti-dental caries properties, with various pharmacological activities such as anti-cancer, skin protection, and hepatoprotective and neuroprotective regulation of gut microbiota, antioxidant, cardio-protectant, and nephroprotective effects. Among all TFs, TF3 is especially studied, largely for its biological effects such as antioxidant, anti-inflammatory, anti-cancer, and anti-microbial activities. Also, synergistic activity of TFs was reported when used in combination with drugs [44]. Among many properties, TFs have been shown to activate caspase 3, inhibiting the MAPK and activating the AMPK pathway, regulating NO signaling, scavenging of radical oxygen species, preventing cell-mediated LDL oxidation, and down-regulating the KIM-1 level. A large number of *in vitro* and *in vivo* studies on the TFs’ effects are reported, but still there are not enough clinical studies available to show their clinical efficacy, although the number of such studies is growing. Also, new formulations (nanoformulation/encapsulation) that are being developed to increase the bioavailability and efficacy of TFs have already shown promising results, which can help in propagation of their use and be recommended for future studies [42–45].

## Material and methods

### Test compounds, inhibitors, bacterial strains, and culture media

*S*. *aureus* USA300 and Wood 46, as well as human primary epidermal keratinocytes, were purchased from (ATCC Manassas, VA). If not specified, all compounds, including stimulatory and inhibitory agents, used in this study were obtained from Sigma (St. Louis, MO). *S*. *aureus* cultures were grown in tryptic soy broth (TSB) or tryptic soy agar (TSA), and, unless otherwise stated, all broth cultures were grown at 37°C. Primary keratinocytes were cultured in Dermal Cell Basal Medium (DCB medium) supplemented with a Keratinocytes Growth Kit from ATCC and grown at 37°C with CO_2_ incubator. Native Hla (active, purified, and unconjugated) was from Creative Diagnostics (Shirley, NY). sHla (soluble Hla, i.e., secreted Hla stripped by filtration and centrifugation from membrane-associated Hla, active) and EV-Hla (membrane-associated Hla, i.e., secreted Hla and separated from the none-membrane associated Hla by filtration and centrifugation, active) were isolated and quantified according to published methodology [46].

### Minimum inhibitory concentration (MIC) study

Tested TFs were examined for minimum inhibitory concentrations (MIC) against *S*. *aureus* USA300 and Wood 46 strains. Clinical Laboratory Standards Institute (CLSI) and M100 guidelines for microtiter broth dilution testing were followed [47, 48]. Control included only the vehicle (0.02% DMSO). All concentrations were tested in triplicate and repeated three times on different days. Briefly, overnight bacterial cultures were standardized by optical density (OD) to 5 x 10^5^ CFU/ml. Serial dilutions of TFs were performed to achieve a test range of 100–3.125 μg/ml. Tubes were incubated at 37°C for 24h under static conditions. Plates were read at an OD_600_ in a multimode plate reader (Tecan Group Ltd., Switzerland) at 0 and 24h post-inoculation. The following formula, which considers the impact of extract color and vehicle on the OD, was used as previously described [49]. MIC values were assigned based on the concentration, respectively.

### Minimum bactericidal concentration (MBC) study

Tested TFs were examined for minimum biocidal concentrations (MBC) against *S*. *aureus* USA300 and Wood 46 strains. Clinical Laboratory Standards Institute (CLSI) and M100 guidelines for microtiter broth dilution testing were followed [47, 48]. Controls included the vehicle (0.02% DMSO). All concentrations were tested in triplicate and repeated three times on different days. Briefly, overnight bacterial cultures were standardized by OD to 5 x 10^5^ CFU/ml. Serial dilutions were performed to achieve a test range of 100–3.125 μg/ml. Plates were incubated at 37°C for 24h under static conditions. Next, 100 μl of each culture was plated on TBS agar plates for the next 24h at 37°C under static conditions. Colonies were counted and MBC values were assigned based on the concentration, respectively.

### Hemolysis assay

The hemolysis assay was performed as previously reported [50, 51]. Briefly, 100 μl of active recombinant Hla (rHla) with a concentration of 0.5 μg/ml was pre-incubated in 96-well plates in the presence of different concentrations (i.e., 2.5, 5.0, 10, and 25 μg/ml) of TFs at RT for 15 min. Then 100 μl (5 x 10^6^ cells/ml) of defibrinated rabbit erythrocytes (rRBCs) was added to each well. The plates were further incubated at 37°C for 20 min. Triton X-100 (1%) served as the positive control and 100% of hemolyzed rRBCs, while PBS was used as the negative control and as 0% of hemolyzed rRBCs. Control wells received mock treatment of 0.01% DMSO. Following centrifugation, the supernatants were removed, and their OD was measured at 540 nm. Data are presented as a % of control without TFs addition (mean +/- SD, n = 6).

### Hemolytic activity assay

The hemolytic activity assay was performed as previously reported and assessed by measuring the hemolytic activity of culture supernatants on rRBCs [50, 51]. Overnight culture of *S*. *aureus* USA300 strain was filtrated and inoculated at 1:500 into 5 ml of TSB containing TF3 at concentrations of 2.5, 5.0, 10, and 25 μg/ml, followed by incubation at RT for 15 min. Next, 50 μl of each sample was dispensed in quadruplicate into 96-well plates. Then 1% v/v rRBCs (Hemostat Laboratories, Dixon, CA), were added to the microtiter plates at 50 μl per well (yielding a final erythrocyte concentration of 0.5%). After 2h of incubation at 37°C for 20 min., hemolysis was assessed by measuring OD_540_ using a multimode plate reader (Tecan Group Ltd., Switzerland). Triton X-100 (1.0%) served as the positive control and 100% of hemolyzed rRBCs, while 1 x PBS was used as the negative control and as 0% of hemolyzed rRBCs. Control wells received treatment of 0.01% DMSO and 0.5 μg/ml Hla. Data are presented as a % of control without TFs addition (mean +/- SD, n = 6).

### Oligomerization assay

Oligomerization assay was performed as previously reported [52, 53]. Briefly, 20 μg of rHla treated with or without TF3 was incubated with 5 mM deoxycholate at 22°C for 20 min. Next, the mixtures were mixed with 4 × loading buffer without β-mercaptoethanol and incubated at 55°C for 10 min. 25 μl of this reaction mixtures were loaded to 10% PAGE followed by western blot analysis.

### Surface Plasmon Resonance (SPR) binding assay

To determine binding affinity of native Hla with TF3, SPR binding assay was performed (Creative Biostructure, Shirley, NY). The *S*. *aureus* Hla was printed onto the chip, while TF3 was the analyte. After chip activation, ligand printing and chip blocking programs were performed, which resulted in *S*. *aureus* Hla automatically printing onto the CM5 chip (Cytiva, Marlborough, MA). Next, the Biacore system pipeline was washed with HBS-EP buffer, and various concentrations of TF3 were loaded into a Biacore 3000 injector (Cytiva, Marlborough, MA). The program was run with the flow rate = 30 μl/min. to determine the binding affinity between analytes and ligands. All the measurements were performed at 25°C. The signal changes (in AU) after binding and washing were recorded as the assay value. Selected protein-grafted regions in the SPR images were analyzed, and the average reflectivity variations of the chosen areas were plotted as a function of time. Real-time binding signals were recorded and analyzed by Data Analysis Module (DAM, Plexera Bioscience, Seattle, WA). Kinetic analysis was performed using BIA evaluation 4.1 software (Biacore Inc., Marlborough, MA). After data collection, kinetics fitting, and data analysis, the KD was calculated.

### *In vivo S*. *aureus* skin infection study

8-10-week-old BALB/c mice weighing approximately 25–30 g were used in this study (Charles River, Wilmington, MA). The mice were kept at an ambient temperature of 21°C with standard rodent diet and water provided *ad libitum* during a light and dark cycle of 12h strictly in accordance with the Animal Welfare Act and the DHHS *Guide for the Care and Use of Laboratory Animals*. Experimental animal protocol No. 03/B012022 was reviewed and approved in 2022 by the Animal Care and Use Committee at the Dr. Rath Research Institute, and all experiments involving animals accurately followed this protocol. Veterinary care was under the direction of full-time veterinarian boarded assistance. BALB/c mice were allowed to acclimatize to the BSL-2 level animal housing facility for seven days, prior to their inclusion in this study, and were then randomly divided into experimental groups as presented in Table 1. Inoculum of 5.0 μg native Hla, sHla, and EV-Hla, respectively, were injected intradermally. Control animals received mock injection of 0.05% DMSO or 50 μg/ml TF3 only. At the end of the experiment (i.e., 48h post-injection), mice were sacrificed by overdosing of isoflurane, skin tissue was carefully shaved with an Accu-Edge microtome blade (Sakura-Finetek, Torrance, CA) and cleansed by wiping with an alcohol prep pad (Covidien, Mansfield, MA). The skin tissues were imaged, collected, and either subjected to permeability assays or immediately snap-frozen in liquid nitrogen and then subjected to western blot. For histology, tissues were fixed in 10% neutral-buffered formalin and stored until standard hematoxylin and eosin (H&E) staining was performed. Stained slides were scanned with the Aperio AT2 system (Leica, Buffalo Grove, IL) and analyzed. The body weights of the mice were measured at the beginning and the end of the experiment. Mice were monitored daily for signs of any distress (i.e., the pre-established benevolent endpoint criteria, e.g., weight loss exceeding 20% of body weight compared to the one recorded on day 0, hunching, loss of mobility and ruffled fur). No such signs of distress or mortality were observed in the present study. All efforts were made to minimize animal suffering. Animal research presented here complied with the ARRIVE guidelines.

**Table 1:** Experimental animal groups of *in vivo* study.

Group	Treatment
1	animals intradermally injected with 0.05% DMSO, n = 4
2	animals intradermally injected with 50 μg/ml TF3, n = 4
3	animals intradermally injected with 5.0 μg/ml EV-Hla, n = 8
4	animals intradermally injected with 5.0 μg/ml sHla, n = 8
5	animals intradermally injected with 5.0 μg/ml native Hla, n = 8
6	animals intradermally injected with 5.0 μg/ml EV-Hla and 50 μg/ml TF3 applied topically every 6h starting 5 min. after Hla injection, n = 8
7	animals intradermally injected with 5.0 μg/ml sHla and 50 μg/ml TF3 applied topically every 6h starting 5 min. after Hla injection, n = 8
8	animals intradermally injected with 5.0 μg/ml native Hla and 50 μg/ml TF3 applied topically every 6h starting 5 min. after Hla injection, n = 8

### Histology study

Skin tissues from mice euthanized on the last day of experiment (i.e., 48h post-injection) were immediately fixed after harvesting in 10% buffered formalin, embedded in paraffin, sectioned (5 μm sections), mounted on glass slides, and stained with H&E (standard histological routine was followed) that was performed cryptically coded sections by third party at the Inotiv facility (Boulder, CO).

### Tissue permeability assay

Two permeability assays, Evans blue (EB) penetration, and fluorescein-labeled ovalbumin (OVA) penetration, were performed according to the described method [54]. For Evans blue dye assays and fluorescein-labeled OVA penetration, 5 μg native Hla was used (see Table 1). Control mice received injection of 1 x PBS or 0.05% DMSO. For EB assay, after 48h, 0.1% Evans blue was added for 10 min. on dorsal skin, followed by washing with 1 x PBS. Skin was then excised, immersed in formamide, and incubated at 60°C. After 6h, OD_620_ nm was measured. For OVA-fluorescein penetration, 50 μg of fluorescein-conjugated OVA were added twice and the skin was excised and homogenized. Fluorescence was measured at Ex/Em = 488/530 nm using a multimode plate reader (Tecan Group Ltd., Switzerland). Data are expressed as a % of control without TF3 addition (mean +/- SD, n = 6).

### NFκB activity assay

NFκB activity was performed using Indigo Biosciences assay kit (State College, PA). Per instruction, a 21 ml suspension of NFκB reporter cells was dispensed into a 96-well plate (200 μl per well) and pre-incubated at 37°C/5% CO_2_ for 6h. Next, the medium was removed, and cells were rinsed with cell screening medium (CSM) followed by its replacement with stock solution of TF3 diluted with CSM to concentrations ranging between 12.5–50 μg/ml. Phorbol 12-myristate 13-acetate (PMA) at 3 nm served as positive control, and 0.05% DMSO as a ‘no treatment’ control was included. The plates were transferred into a culture incubator for 23h. Afterward, the treatment medium was discarded and 100 μl of luciferase detection reagent (LDR) was added to each well of the assay plate. After 5 min., chemiluminescence was measured. Data are expressed as a % of control without TF3 addition (mean +/- SD, n = 4).

### Cytotoxicity assays

Cytotoxicity of TF3 (i.e., 5–50 μg/ml) with and without Hla (0.5 μg/ml native Hla, 0.5 μg/ml sHla or 0.5 μg/ml EV-Hla) was evaluated with the MTT Viability/Cytotoxicity Assay Kit (Cayman, Ann Arbor, MI), LDH Cytotoxicity assay (Cayman, Ann Arbor, MI), and Annexin V Apoptosis assay (Abcam, Fremont, CA) on human primary keratinocytes. For that purpose, keratinocytes were seeded as 5 x 10^4^ cells/well in 96-well tissue culture plates. Plates were incubated for 16h, allowing attachment, prior to treatment. Next, different concentrations of TF3 alone or together with Hla were added, and plates were incubated at 37°C for 24h, followed by the manufacturer’s protocol for detecting cell viability. Control wells received mock treatment of 0.05% DMSO. Data are presented as a % of control without TF3 addition (mean +/- SD, n = 8).

### ELISA assay

Hla was quantified using ELISA assay performed according to previous report [55]. Briefly, 96-well plates were coated with 1 mg/ml Hla antibody and incubated overnight at 4°C. Next, the plates were washed three times with 1 x PBS with 0.05% Tween-20 and blocked with 2% (w/v) non-fat dry milk in 1 x PBS for 2h at 37°C. The blocked plates were washed again, and filtrated media from *S*. *aureus* USA300, treated previously with different concentrations of TF3 (i.e., 5–50 μg/ml), was added, and again incubated, at RT, for 2h. After washing, anti-mouse IgG, conjugated to horseradish peroxidase (HRP; KPL Inc., Gaithersburg, MD) at 1:1000 dilution, was added to the plates, and incubated at RT for another 2h. Plates were then washed again, and signal was analyzed by adding ABTSH ELISA HRP Substrate (KPL Inc. Gaithersburg, MD). The reaction was stopped with ABTSH Stop Solution (KPL Inc. Gaithersburg, MD), and OD_450_ with microplate reader (Molecular Devices, Sunnyvale, CA). Data are presented as a % of control without TFs addition (mean +/- SD, n = 6).

### qPCR analysis

*S*. *aureus* USA300 were first treated with 4 μg/ml lysozyme in TE buffer for 1h at 37°C. Bacterial RNA was next isolated and purified using Total RNA Purification Kit (Norgen Biotek, Thorold, ON) according to the manufacturer’s protocol. Thereafter, a total of 100 ng of RNA was reverse transcribed (RT) using Maxima First Strand cDNA Synthesis Kit for qPCR (ThermoFisher Scientific, Waltham, MA) according to the manufacturer’s protocol. A control reaction without RT was set up for each sample to rule out residual DNA. The cDNA synthesis reaction was diluted in water and 1/2000 was used for qPCR. At least three independent RNA preparations were performed. Quantitative PCR Real-time qPCR was completed with the Bio-Rad CFX96 thermocycler (Bio-Rad, Hercules, USA) using the DyNAmo HS SYBR Green qPCR kit (ThermoFisher Scientific, Waltham, MA). Primer sequences for 16S rRNA (i.e., internal or reference control used to normalized expression levels of target genes), *hla* and *agrA* genes (i.e., target genes) were as follow: hla F- 5’-GCAAATGTTTCGATTGGTCA-3’ and hla R—5’-CCATATACCGGGTTCCAAGA-3’, agr F—5’- CGAAGACGATCCAAAACAAAG-3’ and agr R—5’- ATGTTACCAACTGGGTCATGC-3’, 16s RNA F—5′-TTATGGTGCTGGGCAAATACA-3′ and R—5′-CACCATGTAAACCACCAGATA-3′ with cycling parameters as: 20 seconds at 95°C; 40 cycles at 95°C for 3 seconds and at 60°C for 30 seconds as published [34]. Data were calculated according to Livak method [56].

### Western blot (WB)

An overnight culture of *S*. *aureus* USA300 was inoculated into 1 ml of TSB at 1:500 and grown at 37°C with shaking (250 rpm), in the presence of either DMSO or TF3 at concentrations of 12.5, 25, and 50 μg/ml. Following 24h of incubation, 500 μl of each culture was filter sterilized using a cellulose acetate SpinX 0.22 μm filter (Corning Life Sciences, Tewksbury, MA) and the filter-sterilized media was stored at -20°C. The filtered media was electrophoresed on gradient 8–16% SDS-PAGE gels and transferred to PVDF membranes (Bio-Rad, Hercules, CA). Membranes were blocked overnight at 4°C in TBST (20 mM Tris [pH 7.5], 150 mM NaCl, 0.1% Tween 20) with 5% non-fat dry milk, and then washed 3 times with TBST. Hla was detected using a polyclonal rabbit anti-Hla antibody (Shlievert Lab, University of Iowa) at a1:5000 dilution and a goat anti-rabbit HRP secondary antibody (Cell Signaling, Danvers, MA) at a 1:10000 dilution. WB images were acquired using the Azure cSeries 600 system and auto-exposure settings (Azure Biosystems, Dublin, CA).

Human primary keratinocytes were treated with indicated concentrations of TF3, with or without Hla, and lysed using lysis buffer [50 mM Tris-HCl (pH = 7.4), 1.0% Triton X-100, 150 mM NaCl, 1.0 mM EDTA, 2.0 mM Na_3_VO_4_, and 1 X complete protease inhibitors (Roche Applied Science, Indianapolis, IN)]. The protein concentration was measured by the Dc protein assay (Bio-Rad, Hercules, CA). A 50 μg/well of protein was separated on 4–15% gradient SDS-PAGE gels (i.e., tris-based electrophoresis using standard Laemmle’s method) and transferred to a PVDF membrane. Proteins were detected either with anti-E-cadherin antibody at 1:1000 dilution, anti-ZO-1 antibody at 1:1000 dilution, or anti-claudin antibody at 1:1000 dilution (Cell Signaling, Danvers, MA). WB images were acquired using the Azure cSeries 600 system and auto-exposure settings (Azure Biosystems, Dublin, CA).

### Statistical analysis

All data are presented as means ± SD (n = 3). All experiments were performed at least three times each, at least in triplicates. The Student’s two-tailed t test was used to determine statistically significant differences set at 0.05 levels. Statistical analysis was performed using GraphPad software.

## Results

### Bacteriostatic and bactericidal effect of theaflavins and catechins on *S*. *aureus*

**Fig 1:**
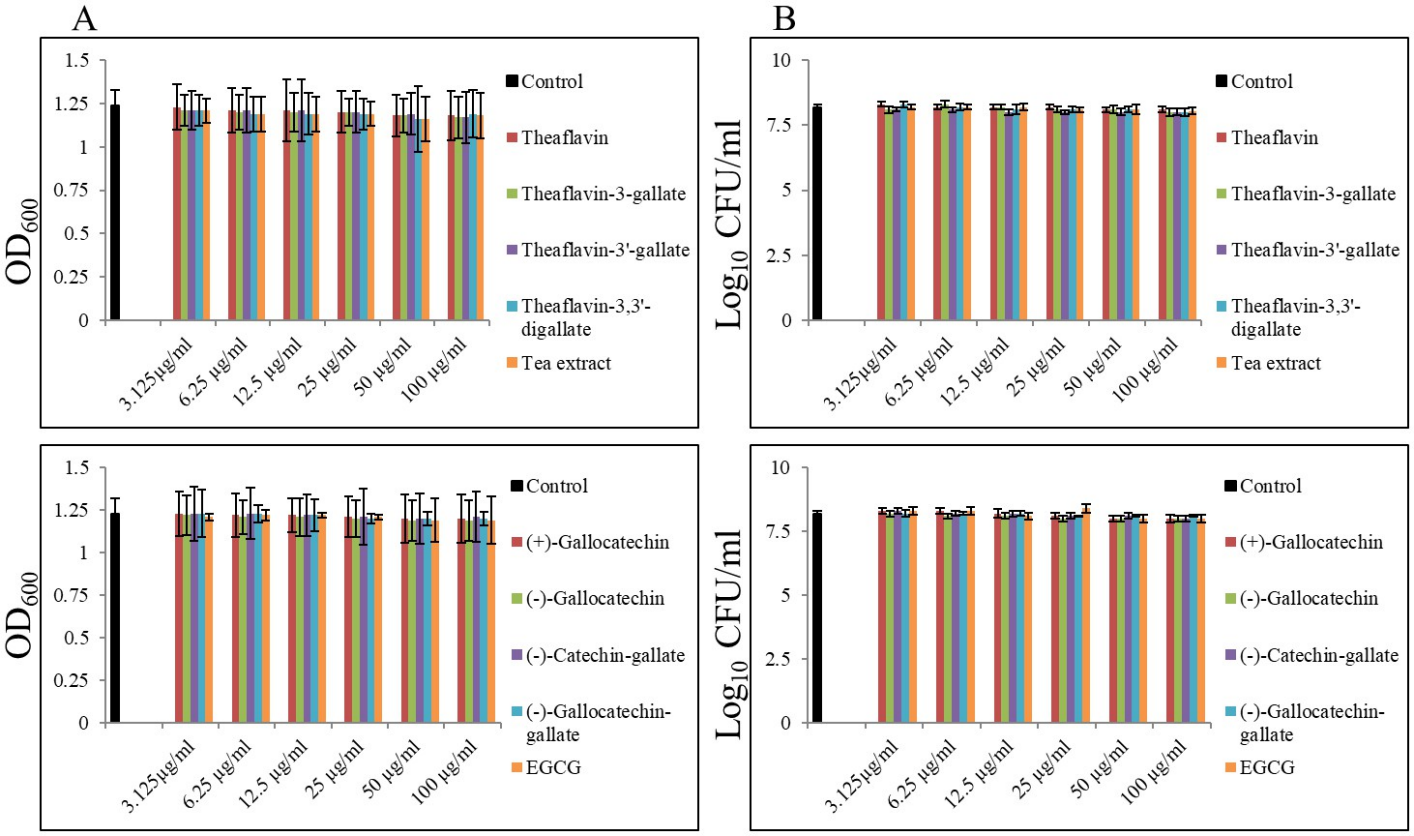
Bacteriostatic and bactericidal efficacy of theaflavins and catechins on *Staphylococcus aureus* USA300. (A) Bacteriostatic effect of theaflavins and catechins was determined by standard macro-dilution assay after 24h. (B) Bactericidal effect of theaflavins and catechins was determined from broth macro-dilution tube test by sub-culturing it to blood agar plates that do not contain the test agent after 24h incubation; control—0.02% DMSO.

Altogether we screened 9 compounds from two main groups of agents derived from *Camilla senesis*, i.e., theaflavins and catechins, in order to check their bacteriostatic and bactericidal effect against MRSA USA300 strain. None of them was able to inhibit growth or have biocidal effect on these tested strains up to 100 μg/ml. We thus were unable to establish MIC_90_ and MBC_90_ values for all tested compounds against MRSA USA300 strain (Fig 1).

### Effect of theaflavins and catechins on production and secretion of Hla from *S*. *aureus*

**Fig 2:**
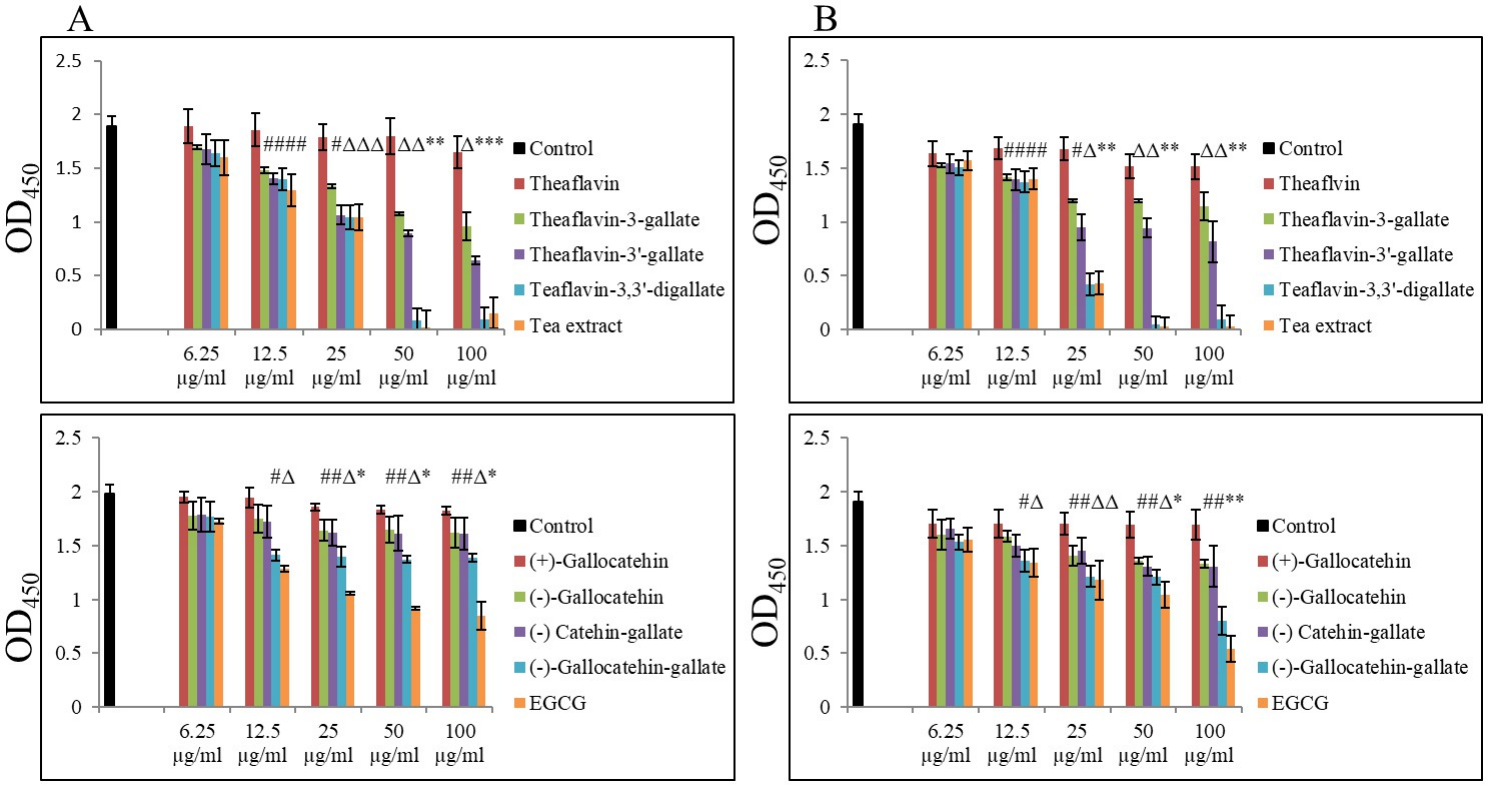
Inhibitory effect of theaflavins and catechins on Hla secretion by *Staphylococcus aureus*. (A) Dose-dependent decrease in Hla secretion by *S*. *aureus* USA300 after 24h treatment with theaflavins and catechins was quantified by ELISA assay. (B) Dose-dependent decrease in Hla secretion by *Staphylococcus aureus* Wood 46 after 24h treatment with theaflavins and catechins was quantified by ELISA assay. Significant differences between treatment and control are represented as # p ≤ 0.05, Δ p ≤ 0.01, * p ≤ 0.001; control—0.02% DMSO; dash line—0.5-fold of change reflecting 50% of expression level of target gene.

**Fig 3:**
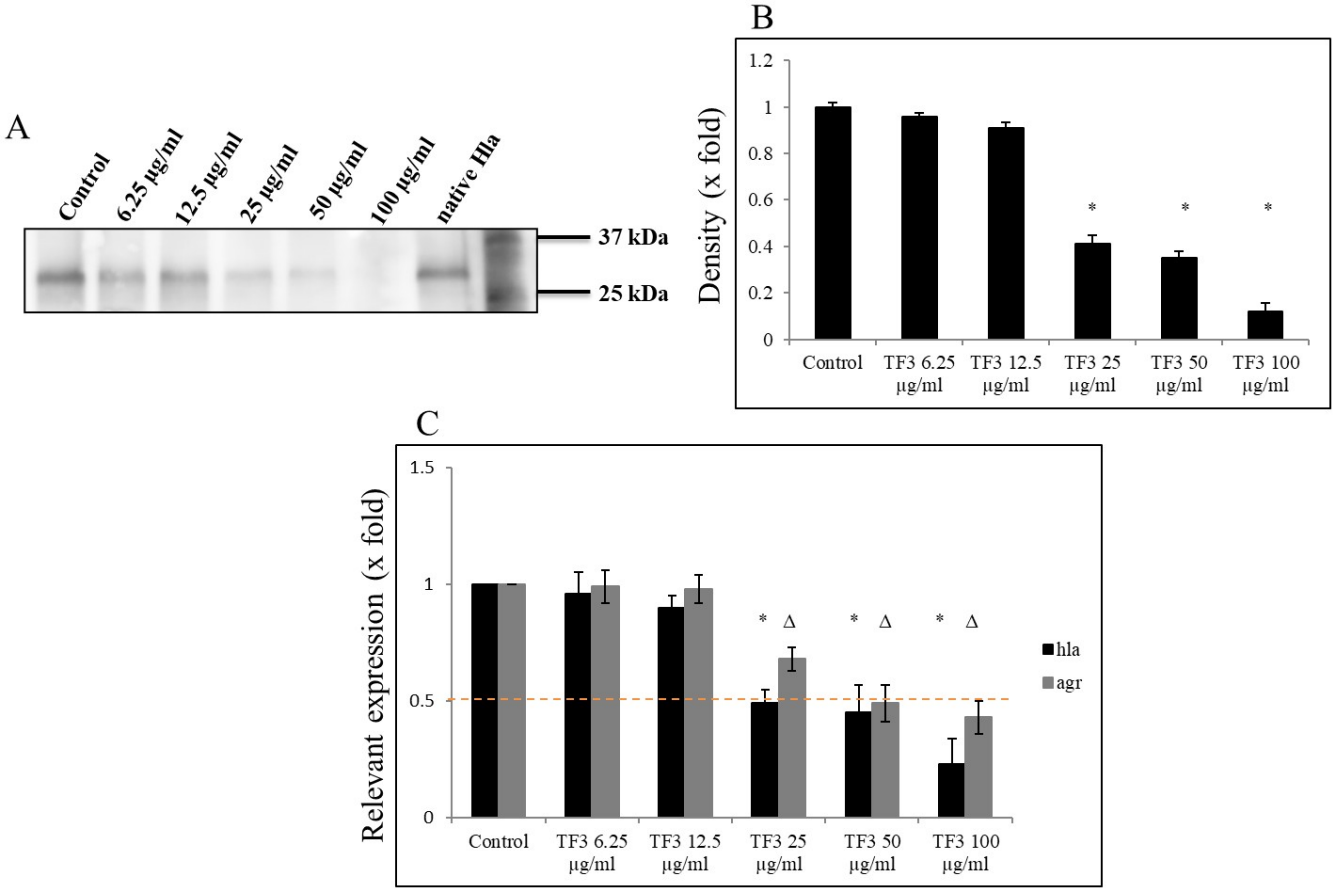
Inhibitory effect of TF3 on Hla production by *Staphylococcus aureus* USA300. (A) Inhibitory effect of TF3 on Hla production. Dose-dependent decrease in Hla protein secretion by *S*. *aureus* USA300 after 24h treatment with TF3 as illustrated by western blot. (B) Densitometry of western blot bands. (C) Dose-dependent decrease in *hla* and *agr*A genes expression by *S*. *aureus* USA300 after 24h of treatment with 12.5–50 μg/ml TF3 assessed by qPCR. Significant differences between treatment and control were assessed by densitometry and are represented as Δ p ≤ 0.01, * p ≤ 0.001; control—0.01% DMSO.

Since no effect on growth and viability of *S*. *aureus* USA300 was observed, we looked at the production and secretion of staphylococcal Hla, as a crucial virulence factor contributing to skin and pulmonary infections. Altogether, we again screened 9 compounds from two groups, i.e., theaflavins and catechins up to 100 μg/ml concentrations. As presented in Fig 2, all theaflavins were more effective in inhibiting secretion of Hla compared to catechins. Also, amongst theaflavins an interesting pattern was observed, as examined by ELISA assay. Namely, that compounds with galloyl residue, such as TF1 and TF2, were more effective in decreasing Hla secretion compared to TF (i.e., compound without this residue), and that the compound with additional galloyl residues, such as TF3, revealed the most inhibitory effect compared to TF, TF1 and TF2, respectively. Western blot analysis further confirmed a dose-dependent inhibitory effect of TF3 on Hla secretion by *S*. *aureus* USA300, with a decrease reaching nearly 95% at 100 μg/ml concentration (Fig 3A and 3B and S1 Fig in S1 Raw images). As TF3 reduced the secretion of Hla, we therefore further checked the expression status of *hla* and *agrA* genes. As shown in Fig 3C, the transcriptional level of these genes was negatively affected by treatment with the same increasing concentrations of TF3 as well. Exposure of *S*. *aureus* USA300 cultures to ≥25 μg/ml of TF3, significantly decreased the *hla* gene expression by about 0.49-fold (51% decrease in expression) and reached the level of about 0.21-fold (79% decrease in expression) at 100 μg/ml. A similar dose-dependent pattern was observed with inhibition of the *agrA* gene with ≥50 μg/ml of TF3 with 0.36-fold (64% decrease in expression) at 100 μg/ml of TF3.

### Effect of TFs on activity of Hla from *S*. *aureus*

**Fig 4:**
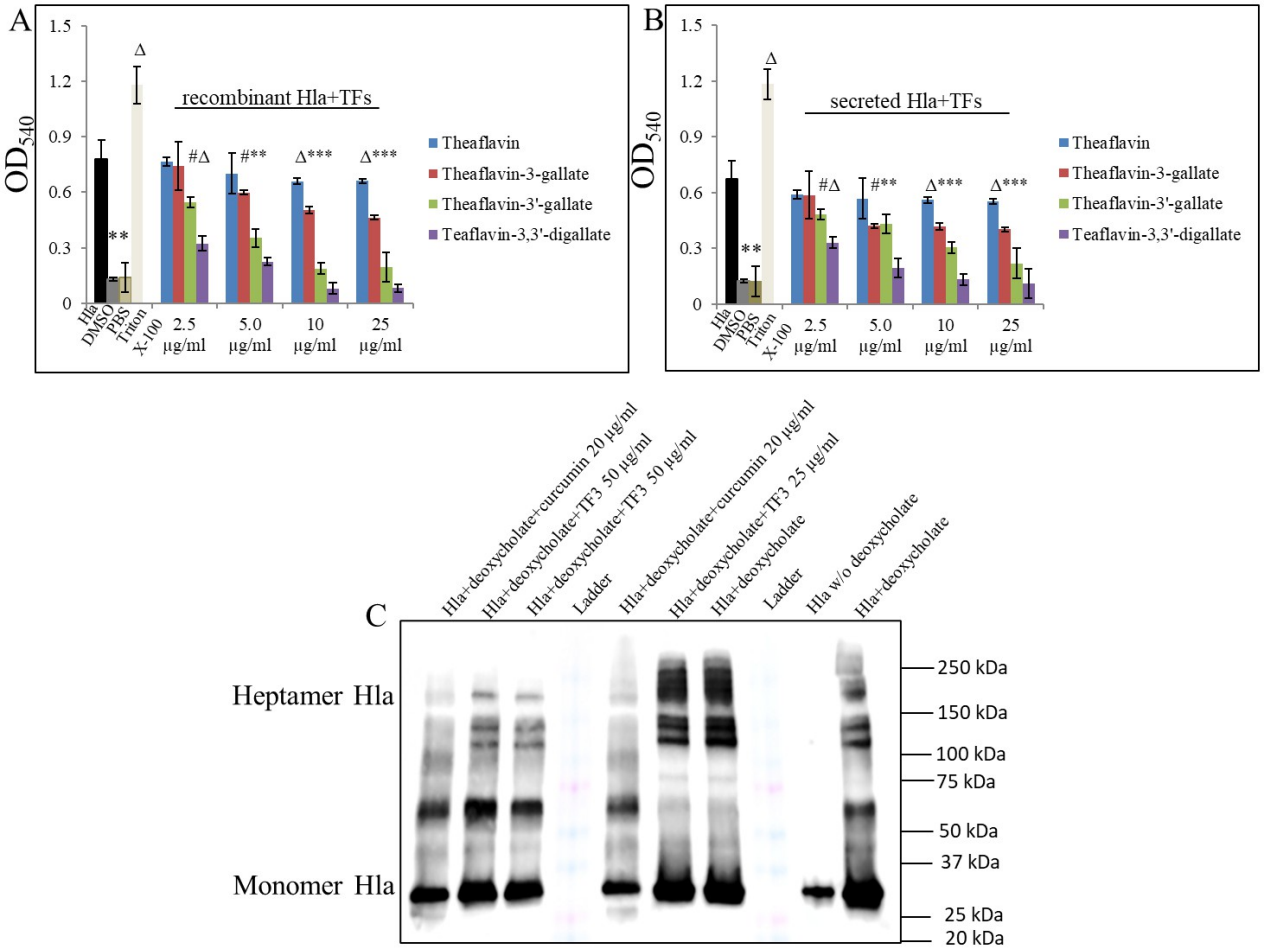
Inhibitory effect of TF3 on Hla activity. (A) Dose-dependent decrease of rHla hemolytic activity by TF3 as demonstrated by rRBC hemolysis assay. 10% rRBCs were exposed to increasing doses of TF3 for 10 min. and treated with 0.5 μg/ml of Hla for 20 min. (B) Dose-dependent decrease of secreted Hla hemolytic activity by TF3 as demonstrated by rRBC hemolysis assay. 10% rRBCs were added to 100 μl of sample containing supernatant from *S*. *aureus* USA300 overnight culture that was supplemented with different concentrations of TF3 and incubated at RT for 10 min. Significant differences between treatment and vehicle are represented as # p ≤ 0.05, Δ p ≤ 0.01, * p ≤ 0.001; control sample—0.5 μg/ml rHla or 100 μl of supernantant+0.01% DMSO, negative controls—1 x PBS or 0.01% DMSO, positive control—1.0% Triton X-100. (C) Effect of TF3 on Hla oligomerization. Dose-dependent decrease in 5 mM deoxycholate-induced oligomerization of 20 μg/ml native Hla was assessed by western blot, controls—0.01% DMSO+native monomeric Hla treated with or without 5 mM of deoxycholate.

To further elucidate the biological relevance of TFs on Hla, we sought to determine whether TFs impair its hemolytic activity as well. Thus, we first exposed rHla to increasing concentrations of TFs and performed a hemolysin release assay (Fig 4A). We used rabbit red blood cells (rRBCs), since it was reported that Hla very effectively hemolyzes them [50]. Our results confirmed that observation and further revealed that treatment of rHla with increasing concentrations of TFs significantly inhibits its lytic activity. The results affirmed a previously observed pattern showing that the more galloyl residues in the compound, the stronger its anti-hemolytic activity, thus TF3 was shown to be the most effective inhibitory compound among all tested TFs, which at as low as 2.5 μg/ml concentration already significantly inhibited heme release in 62.5%, with 100% blocked heme release at its 10 μg/ml concentration. In terms of hemolytic activity, where we used secreted Hla instead of rHla, we also found that hemolysis was significantly inhibited by TF3 (Fig 4B). The results showed the 52.9% inhibition of secreted Hla activity at 2.5 μg/ml TF3 concentration, and 100% observed inhibition at 10 μg/ml TF3. In the absence of TFs, both rHla and secreted Hla caused almost complete lysis of rRBCs (control samples), contrary to the samples containing only 1 x PBS (negative control). Consistent with the results obtained thus far, we could demonstrate that TF3 significantly inhibits the hemolytic activity of Hla of *S*. *aureus* USA300 at concentrations lower than the ones that affect Hla’s expression.

### Effect of TF3 on oligomerization of Hla from *S*. *aureus*

Another aspect of Hla’s biological role is its oligomerization (heptamer formation) as being critical for the pore-forming activity of the protein. Thus, an oligomerization assay was employed to further evaluate whether TF3 treatment could interfere with the formation of Hla oligomers. When the monomeric Hla (33.2 kDa) was incubated with deoxycholate, in order to induce its oligomerization [52], a stable heptamer (Hla7, 232.4 kDa) was pictured (Fig 4C and S2 Fig in S1 Raw images). Increasing concentrations of TF3 did not, however, significantly inhibit formation of a stable heptamer induced by the deoxycholate, thus implying direct interaction between Hla and TF3 that subsequently affects activity of Hla exclusively.

### Determination of the binding parameters of Hla with TF3

**Fig 5:**
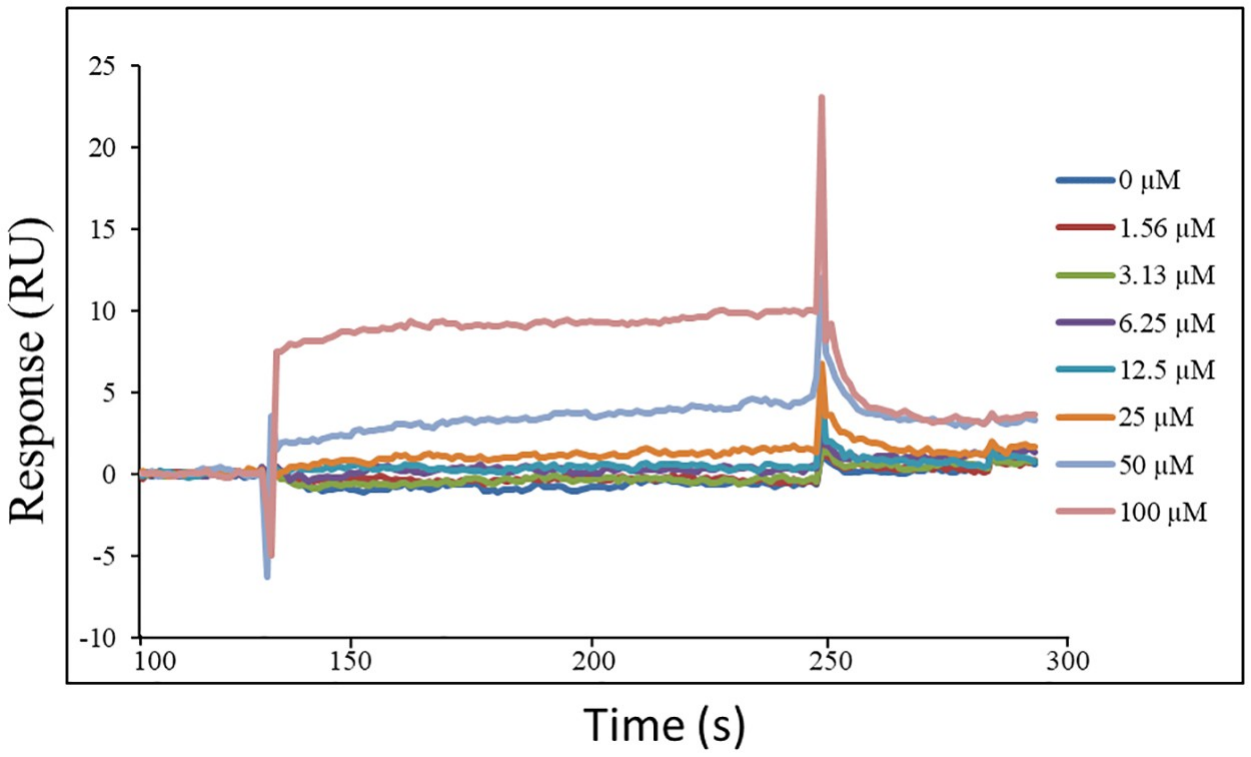
Binding of TF3 to Hla of *S*. *aureus*. SPR result of Hla with TF3. Sensorgram curve of TF3 with concentration of 0 μM, 1.56 μM, 3.13 μM, 6.25 μM, 12.5 μM, 25 μM, 50 μM, 100 μM binding to Hla for evaluation of the binding affinity and affinity parameters such as KD, ka and kd.

To better understand the mechanism of the action of TF3, we experimentally validated its Hla-binding behavior in SPR assays and evaluated its binding affinity according to the steady-state analysis fitting curve (Fig 5). By completing the SPR binding assay the association rate constant Ka as 4.43×10^2^ M^-1^s^-1^ and the dissociation rate constant Kd as 2.03×10^−2^ s^-1^ was determined. The equilibrium dissociation constant (KD) as 4.57×10^−5^ M indicates that TF3 possesses an ability to bind Hla with a moderate affinity.

### Effect of TF3 on Hla-induced cytotoxicity in human keratinocytes

**Fig 6:**
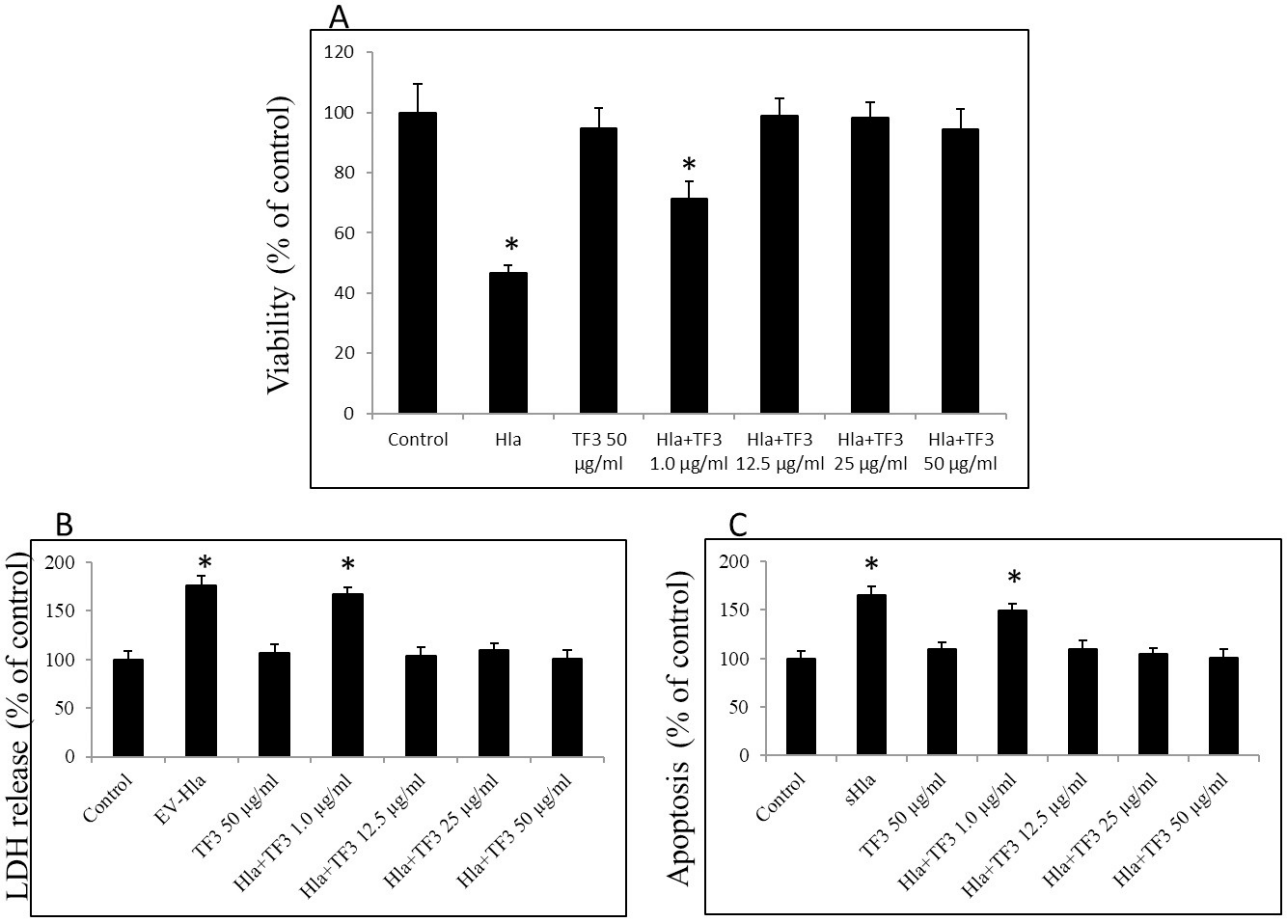
Cytotoxic effect of Hla and TF3 on human primary keratinocytes. (A) Cells were treated with native Hla at 0.5 μg/ml concentration alone or with different concentrations of TF3 in order to check the survival rate that was determined using MTT method and measuring absorbance at 570 nm after 24h. (B) Cytotoxic effect of EV-Hla at 0.5 μg/ml concentration alone or with different concentrations of TF3 on cells determined using LDH method and measuring absorbance at 450 nm after 24h. (C) Viability of cells treated with sHla at 0.5 μg/ml concentration alone or with different concentrations of TF3 in order to check the their apoptosis status determined using Annexin V method and measuring fluorescence Ex/Em = 488/530 nm after 24h. Significant differences between treatment and control are represented as * p ≤ 0.001; control—0.05% DMSO.

Hla of *S*. *aureus* is reported to be more recurrent in patients with skin infections, including *atopic dermatitis* (AD), as well as being highly cytotoxic towards several cell types, including keratinocytes [35, 57–59]. Based on the aforementioned findings, we further evaluated the ability of TF3 to protect keratinocyte cells from Hla-mediated injury. The effect on human primary epidermal keratinocyte survival was assessed by measuring cell viability after treatment with 0.5 μg/ml native Hla. The results corroborated previous reports about Hla’s significant cytotoxic effect towards keratinocytes. Compared to untreated cells, viability of keratinocytes assessed by MTT dropped to 42.2% after treatment with native Hla, whereas treatment with increasing concentrations up to 50 μg/ml of TF3 was positively associated with their survival rate. Compared to untreated cells, the survival rate of keratinocytes co-treated with native Hla and 12.5 μg/ml of TF3 increased to 90.8%, while the survival rate of keratinocytes co-treated with native Hla and 25 μg/ml of TF3 increased to 90.6%, and to 89.8% when co-treated with native Hla and 50 μg/ml of TF3 (Fig 6A). Data obtained here and by us previously have consequently demonstrated that TF3 did not affect the cell viability at up to 50 μg/ml concentration [60]. Thus, our result indicates that TF3, at the concentrations of 12.5 to 50 μg/ml, was able to protect keratinocytes from injury in a dose-dependent manner.

As previously reported, different forms of Hla have differential cell-death triggering mechanism. As shown, EV-Hla causes their necrosis of keratinocytes, whereas sHla induces their apoptosis [54]. Thus, to further validate our results, we checked the level of lactate dehydrogenase (LDH) released into culture media of EV-Hla-treated keratinocytes (necrosis marker), and annexin V-positive keratinocytes (apoptosis marker) after treatment with sHla. In accordance with the previously reported observations, the morphological changes of keratinocytes in the form of swallowing, detachment, and rupture, were noticeably decreased after TF3 treatment in contrast to the Hla treatment [54]. The cytotoxic effect on human primary epidermal keratinocyte was elevated significantly after treatment with 0.5 μg/ml EV-Hla and 0.5 μg/ml sHla, respectively. Keratinocyte viability was noticeably decreased upon treatment with EV-Hla and accounted for a 76.3% increase in released LDH, as well as a 65.3% increase in apoptotic cells, compared to their respective controls. Again, treatment with increasing concentrations up to 50 μg/ml of TF3 have a protective effect on keratinocytes. Co-treatment of EV-Hla with TF3 (at the concentrations of 12.5 to 50 μg/ml) did not augment levels of LDH, neither did sHla co-treated with TF3 (at the concentrations of 12.5 to 50 μg/ml) increase annexin-positive cells. In both cases cytotoxicity was similar to levels of their respective controls (Fig 6B and 6C).

### Effects of TF3 on Hla-induced pro-inflammatory cytokines

**Fig 7:**
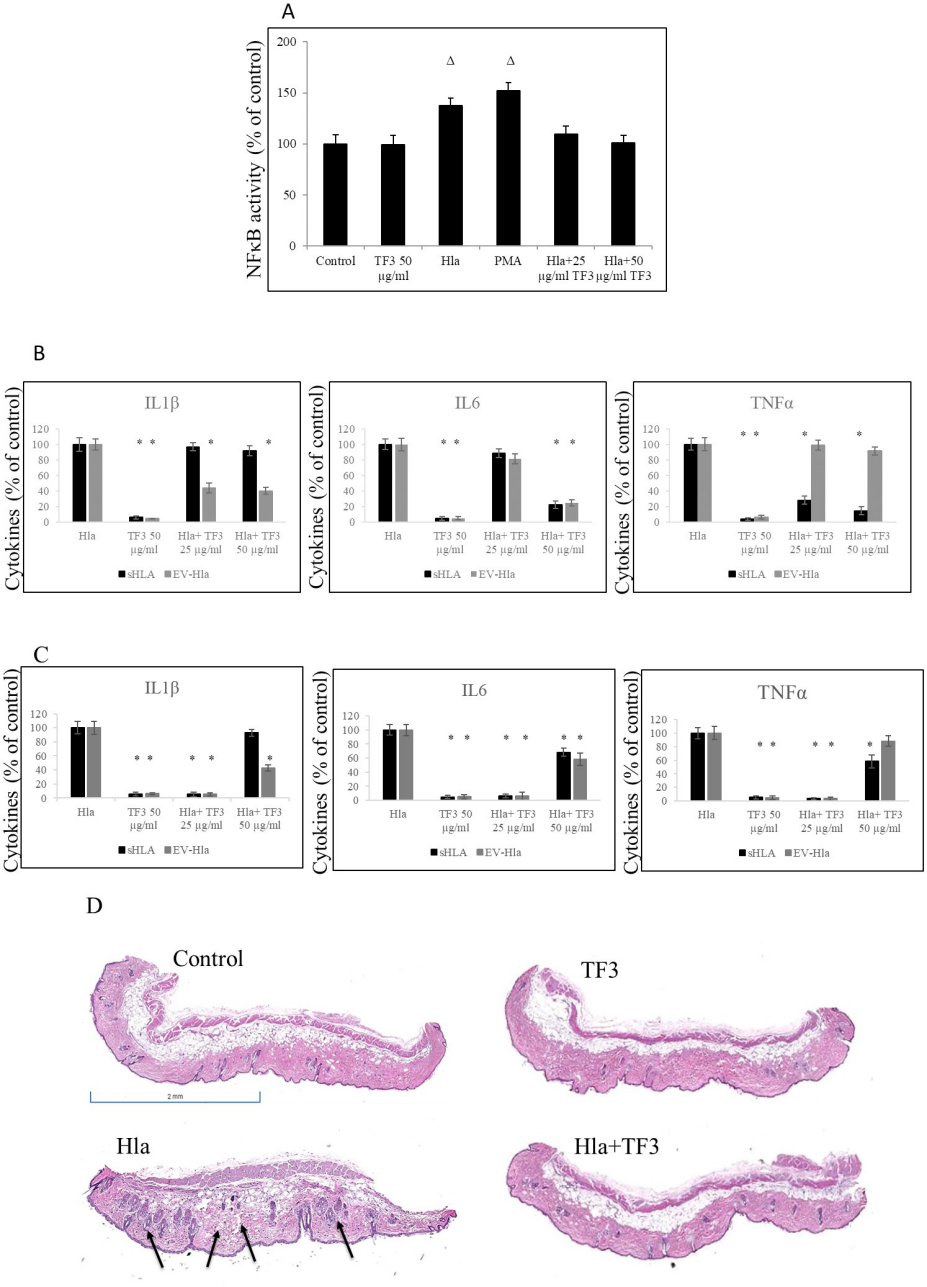
Cytokines status *in vitro* and *in vivo*. (A) Effect of native Hla alone and with TF3 on activity of NFκB performed as described in Material and Method section; control—0.05% DMSO, positive control—control cells stimulated with PMA at 3 nm concentration. (B) Human primary keratinocytes were treated with different concentrations of TF3 upon EV-Hla and sHla stimulation, respectively. Levels of secreted pro-inflammatory cytokines were assessed by ELISA after 24h post-treatment. (C) Mouse skin tissue samples injected with native Hla at 5.0 μg/ml concentration alone or with 50 μg/ml TF3. Levels of secreted pro-inflammatory cytokines were assessed by ELISA after 48h. (D) Images or representative skin alterations after treatment with 5.0 μg of native Hla alone together with different concentrations of TF3 for 48h stained with H&E; black arrows—noticeable skin pathology as described in Result section, control animals treated with DMSO only n = 4, control animals treated with TF3 only n = 4, animals treated with Hla only n = 8, animals treated with Hla and TF3 n = 8. Significant differences between treatment and control are represented as # p ≤ 0.05, Δ p ≤ 0.01, * p ≤ 0.001.

Previous reports have shown that Hla of *S*. *aureus* induces pro-inflammatory cytokine production by several cell types, such as dermal fibroblasts, airway epithelial cells, and keratinocytes [35, 51, 53, 54, 57–59]. When we used NFκB reported cells in order to check whether TF3 affects the activity of NFκB, an archetypal proinflammatory signaling pathway, we were able to show that, indeed, TF3 application decreases the activity of NFκB reported cells at 25 and 50 μg/ml concentrations to the levels similar to those of its control (Fig 7A). Aforementioned data also showed that EV-Hla is a key player in the development of AD phenotypes, and that there is a difference in cytokines secretion patterns induced by EV-Hla vs. sHla. Collectively, these findings demonstrated that EV-Hla induces IL1β in keratinocytes but inhibits TNFα production in the contrast to sHla, which prompts TNFα production, but that both EV-Hla and sHla enhance IL6 production. We therefore evaluated the effect of TF3 on these inflammatory cytokines relevant in AD pro-cytokines from human primary keratinocytes after treatment with equal but respective amounts of EV-Hla and sHla. We found that the cytokine production profiles indeed differ, but TF3 showed inhibitory effect. In our *in vitro* study, as shown in Fig 7B, when EV-Hla was used as a stimulator, TF3 at 50 μg/ml inhibited secretion of IL6 by about 63.2% and IL1β by about 58.1%, with no significant influence on TNFα level. When sHla was used as a stimulator instead, TF3 at 50 μg/ml inhibited secretion of IL6 by about 65.2% and TNFα by about 54.3%, with no significant influence on IL1β level.

Our *in vivo* results on cytokines status in skin samples of mice intradermally injected with the same amounts of EV-Hla and sHla, respectively, and evaluated by ELISA, corroborated previously observed patterns. In skin samples of mice injected with EV-Hla, elevated levels of IL6 and IL1β were found, compared to either control (i.e., uninfected mice) or the skin samples injected with TF3 only. Following treatment with 50 μg/ml TF3, the percentage of IL6 diminished to 60.4% and IL1β to 43.5%. Similarly, in skin samples of mice infected with sHla, higher levels of IL6, IL1β, and TNFα were present, compared to either control (i.e., uninfected mice) or the skin samples injected with TF3 only. In contrast, in skin lysates treated with 50 μg/ml TF3, the percentage of IL6 was reduced to 78.1%, IL1β to 59.4%, and TNFα to 63.2% (Fig 7C). Moreover, as shown in Fig 7D, histological evaluation of H&E-stained skin sections from mice injected with native Hla and treated 50 μg/ml TF3 showed minor pathological manifestations determined by infiltration of leucocytes being at the level compared to control samples treated with 50 μg/ml TF3 only. The analyzed slides also showed negligible impact of TF3 only, in contrast to samples treated with native Hla only, where visible inflammatory process and less dense dermis was detected.

### Effects of TF3 on Hla-induced skin barrier disruption

**Fig 8:**
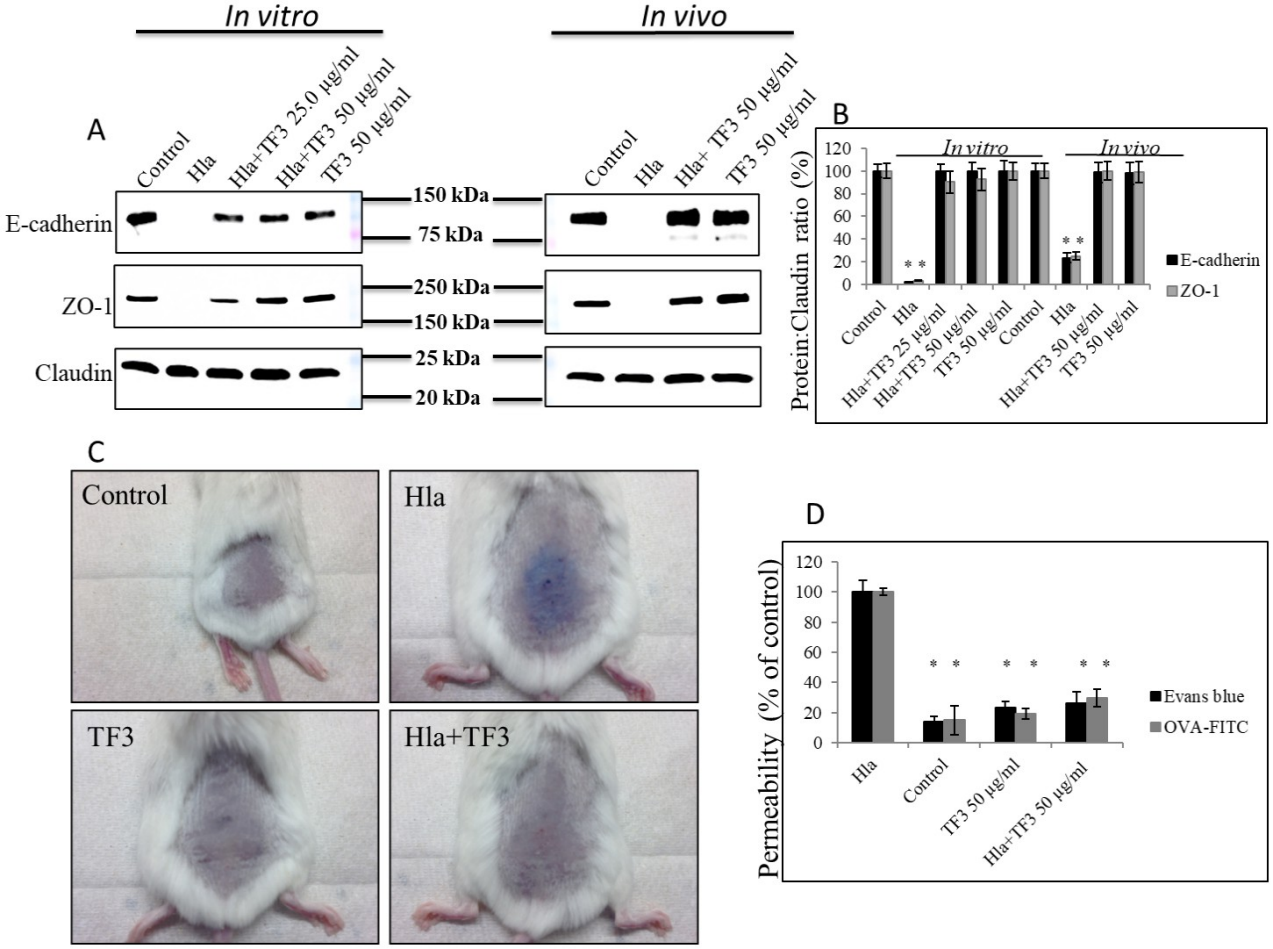
Effect of Hla and TF3 on skin barrier. (A) Status of key molecules of adherence and tight junctions at the cellular level analyzed by western blot using antibodies against E-cadherin, ZO-1, and claudin. (B) Densitometry of western blot bands. (C) Representative images of mouse skin after Evans blue dye penetration upon treatment with native Hla at 5.0 μg/ml concentration alone or together with 50 μg/ml concentration TF3 (see Table 1). (D) Quantification of Evans blue dye and fluorescein-labeled OVA penetration into mouse skin after treatment with native Hla at 5.0 μg, respectively. Significant differences between treatment and control are represented as Δ p ≤ 0.01, * p ≤ 0.001; control—0.05% DMSO.

After information obtained from analysis of H&E slides and seen protective effect of TF3 against Hla-mediated injury of human keratinocytes (resulting in inflammation), we prompted further analysis, to establish whether similar protective effects take place in the skin barrier, known to be affected by Hla as well and having consequences in skin-related infections caused by *S*. *aureus*. To explore this matter, we co-treated human primary keratinocytes with native Hla and either 25 μg/ml or 50 μg/ml concentration of TF3, checking the status of E-cadherin, ZO-1, and claudin after 24h. We noticed that claudin is not affected by Hla or TF3, and others have reported that Hla also does not negatively affect β-catenin level [55, 56]. However, we noticed about 99% decrease in E-cadherin levels after treatment with native Hla only, in contrast to either co-treatment of native Hla with both concentrations of TF3, or controls (i.e., untreated cells or cells treated with TF3 only), which showed no significant changes in its levels. A similar effect was obtained with ZO-1. Its expression was significantly affected and accounted for about 98% decrease after treatment with native Hla only, with no observed changes in its levels after co-treatment with 25 μg/ml and 50 μg/ml concentrations of TF3 (Fig 8A and 8B).

Our *in vivo* experiment, where mice were intradermally injected with 5 μg native Hla, following treatment with 50 μg/ml TF3, showed significant protection after 48h of skin barrier as well, with no mortality occurring and no significant differences between the treatment groups with or without the DMSO as a vehicle, indicating that DMSO has no toxic effect on the infected mice (Fig 8A–8D, S3 Fig in S1 Raw images). We found about 87% elevated level of Evans blue and about 86% elevated level of fluorescence-labeled ovalbumin (OVA) in skin samples of mice injected with native Hla, compared to control samples (i.e., untreated or treated with TF3 only). In skin samples co-treated with native Hla and TF3, these levels were significantly lower and accounted for about 75% (Evans blue) and 73% (OVA), compared to samples treated with native Hla only (Fig 8C). Moreover, western blot analysis of skin samples revealed 78% decrease of E-cadherin after treatment with native Hla only, in contrast to co-treatment with native Hla and 50 μg/ml TF3, which resulted in no significant changes in E-cadherin expression. ZO-1 expression was negatively affected as well, with 76% decrease after treatment with native Hla only, and no significant differences noticed in samples co-treated with native Hla and 50 μg/ml TF3. Interestingly, neither Hla nor TF3 affected claudin levels at all. These results imply that interference of TF3 with Hla could significantly inhibit Hla pathological effect on skin tissue.

## Discussion

It is widely acknowledged, and well-evidenced, that the *Staphylococcus aureus* USA300 strain is the leading pathological cause of community-associated bacterial infections in the United States, and possesses enhanced virulence even compared to traditional hospital-associated MRSA strains [1–8]. In addition, this pathogen can cause severe or even fatal invasive disease, although the vast majority of USA300-caused infections are those of skin and soft tissue and are immediately not life-threatening. Current research also indicates that the USA300 strain produces and secretes relatively high levels of Hla *in vitro*, with implications of pulmonary and skin-related pathogenesis *in vivo*. Hla-mediated cytotoxicity, with its primary (e.g., apoptosis/necrosis of monocytes/macrophages, death and detachment of endothelial cells) and secondary (e.g., affected phagocytosis, disrupted epithelial and endothelial barrier) consequences, results in the loss of the respective cellular functions, and, conceivably, either direct or indirect pathologies, especially in lung and skin tissues [1–25].

Practice of the global and habitual use of antibiotics has improved the quality and length of life for countless people since their earliest discovery. It is now obvious, however, that the previously floated thought that antibiotics could subjugate most bacterial infectious diseases was flawed, owing to the fact of the increasing emergence of antibiotic-resistant and/or highly virulent strains. The occurrences of MRSA infections associated with high levels of morbidity and mortality, which are incrementally growing, while the antibacterial agents available are limited, even with appropriate doses of antibiotics, prove the point. Abating bacterial virulence factors, rather than following a traditional approach aimed at bactericidal and bacteriostatic activity, has been loudly proposed already as an effective and promising strategy against staphylococcal infections, since preferred use of bactericidal antibiotics led to the proliferation of drug-resistant strains [61–69]. Contrary to conventional antibiotics, which function as biocidal agents, a tactic targeting virulent factors might apply a minor selective pressure, since most of them are not essential to bacterial growth, therefore do not instigate the resistance [61–66]. A strategy focused on targeting virulence may lessen pathogenicity and its consequences, hence applying a milder selective pressure for the development of resistance. Based on already reported results and the data provided in this study, Hla may represent a promising anti-virulence target for the development of novel agents against *S*. *aureus*. Successful examples of applying this strategy aimed at attenuating Hla activity have already been reported. Tabor *et al*. demonstrated use of anti-Hla antibodies, whereas Ragle *et al*. used a modified β-cyclodextrin against *S*. *aureus* pneumonia, and studies by Quave *et al*. established that some natural compounds, such as *Castanea sativa* leaf extracts, can attenuate *S*. *aureus* virulence in a mouse skin infection [12, 49, 70]. Since the *hla* gene is contemporaneous in the genomes of most of *S*. *aureus* strains, it is probable that Hla protein contributes to the severity of *S*. *aureus*-caused infections in humans, a perception that has been confirmed in many animal infection models. Nonetheless, cautious application of either synthetic or natural agents should be taken into consideration as well, since it is a known fact that, although inhibitors such as clindamycin and linezolid inhibit the production of virulence factors produced by *S*. *aureus*, others, e.g., β-lactam and glycopeptide antibiotics, induce Hla, enterotoxins, and toxic shock syndrome toxin-1 production, indicating their harmful action, especially in MRSA infections [4–7, 61–66].

Here we add more information to already broad knowledge about TF3 and its versatile efficacy [42–45]. We were able to show that TF3 represses production, and secretion, and, most importantly, inhibits activity of Hla from *S*. *aureus* USA300 with concentrations ranging from 2.5 to 100 μg/ml. We were also able to show that TF3 significantly suppresses expression of exoprotein genes *hla*, and *agrA* in the MRSA USA300 strain. A virulence factor gene *hla* is controlled by *agr* and *saeR/S*, although *agrA* is a regulator associated with the pathogenesis and biofilm formation of *S*. *aureus* as well [31–34]. We noticed that TF3 reduces production of Hla by *S*. *aureus*, primarily by inhibition of the *hla* gene and in part owing to inhibition of the *agr* gene, although at concentrations 10 times higher than the ones inhibiting activity of Hla protein. MD simulation of Hla with TF3, supported by SPR assay, helped to describe the mechanism of the inhibition of the activity, revealing the detrimental residues around the binding site of TF3 governing this binding process and its specificity. The binding affinity KD between *S*. *aureus* Hla and TF3 was calculated as KD = 4.57×10^−5^ M; however, an important implication of this experiment is the mechanism of the inhibition activity. Concluding analysis of the character of the binding demonstrates that the conformation of the binding in the complexes with TF3 is relatively stable, implying that the TF3 binding is the “active” inhibitory force. We believe this is even more important information, considering data firstly showing that Hla is a “core” genome-encoded toxin in essentially all CA-MRSA strains, secondly proving that alternate expression of virulence molecules “injects” variances in virulence of *S*. *aureus* strains, and thirdly revealing that Hla production correlates with refractory skin colonization in patients with atopic dermatitis [58, 59]. A therapeutic approach directed towards Hla would be relatively broad in scope. We could not, however, show bacteriostatic and bactericidal effect of TF3 towards MRSA or MSSA strains, even at high concentrations. Inhibition of Hla activity at relatively low concentrations of TF3, on the other hand, has a good implication, thanks to the fact that this inhibition of virulence and pathogenesis may be accomplished without posing growth and biocidal inhibitory pressures on *S*. *aureus*. It may also imply that it would not affect the common human cutaneous microbiome and trigger bacterial resistance, since a skin microflora is critical to skin barrier health.

Pore formation and subsequent cellular lysis has been considered as the most salient significance of Hla, owing to subsequent alteration of the cell signaling pathways involved in cell proliferation, inflammatory responses, and cell-cell interactions of epithelial cells, endothelial cells, T cells, monocytes, and macrophages [35, 36, 38]. Inflammation and skin barrier exacerbation is proven to be a concerning factor of almost all *S*. *aureus*-related skin infections, including AD [38–41]. The pathology involves intrinsic factors such as host immune responses, and extrinsic ones such as toxins, e.g., Hla. Hong *et al*. found that most of *S*. *aureus* isolated from the skin of AD patients produced Hla (in soluble as well as EV-associated form) [46]. Interestingly, EV-Hla induced AD-like skin inflammation and keratinocyte necrosis, whereas sHla was found to induce their apoptosis. Moreover, EV-Hla up-regulated the production of IL6, while sHla enhanced the production of TNFα, but both increased the level of IL1β. Since TF3 was able to protect human primary keratinocytes from apoptotic and necrotic death, and at the same time inhibit secretion of all relevant cytokines *in vitro* as well as in the mouse skin infection model (as a general or rudimentary approximation of *S*. *aureus* skin infection in humans), TF3’s inhibitory effect on Hla’s activity finds its biological relevance. Another factor strengthening TF3’s prospect is its effect on tight and adherent junctions, shown by Kwak *et al*. to be directly affected by Hla [27]. In addition, Popov *et al*. identified a cytoplasmic member of the adherent junctions, plekstrin-homology domain containing protein7 (PLEKHA7), as yet another Hla receptor, after disintegrin and ADAM10, and reported that it controls severity of skin infection and lethal pneumonia [69]. Disruption of skin barrier integrity by reducing levels of either junctional proteins (e.g., E-cadherin, ZO-1, ZO-3, PLEKHA7) or the receptors for Hla may underlie other consequences and pathologies such as Ca^2+^ influx or sepsis. Thus, the preventive effect of TF3 seen in our study on epithelial permeability, by shielding of the E-cadherin molecule as a major representative of adherence junctions, and ZO-1 molecule as a significant representative of tight junctions from depletion, could have more profound implications. Although it is arguable whether inhibitors of virulent factors could function as impartial therapeutics, their adjunct or adjuvant application might serve the purpose. Interestingly, Wang *et al*. reported about other polyphenols, namely myricetin and curcumin, as promising inhibitors of staphylococcal Hla, whereas He *et al*. widened the spectrum of natural compounds acting inhibitory on hemolytic activity of Hla about kaempferol and quercetin [53, 71, 72].

In conclusion, we have demonstrated that a natural compound, which does not demonstrate “typical” (i.e., bacteriostatic or bactericidal) antibacterial activity, has a great anti-hemolytic efficacy and pronounced potential as a therapeutic or adjunct agent, owing to its ability to explicitly target virulence of *S*. *aureus*, while being non-toxic to human keratinocytes, with no dermato-pathology. As mentioned above, owing to slower development of antibiotics and faster rising bacterial resistance, targeting bacterial virulence rather and the bacterium itself is not only a new idea but also well-acknowledged and approved strategy for the treatment of bacterial infections. Although abolishing the expression and activity of only one of the proteins does not necessarily always lead to recovery, it may nevertheless significantly reduce pathogenicity and speed the recovery process. Thus, TF3 application might be beneficial wherever Hla is a major determinant of infection.

## Supporting information

S1 File Raw western blot images. (PDF)
