# Supplementary material for: Inhibition of α-hemolysin activity of Staphylococcus aureus by theaflavin 3,3’-digallate
Source: PLoS One. 2023 Aug 31;18(8):e0290904. doi: 10.1371/journal.pone.0290904 (PMC10470925; doi:10.1371/journal.pone.0290904)
Supplement: S1 File — (PDF) [file pone.0290904.s001.pdf]

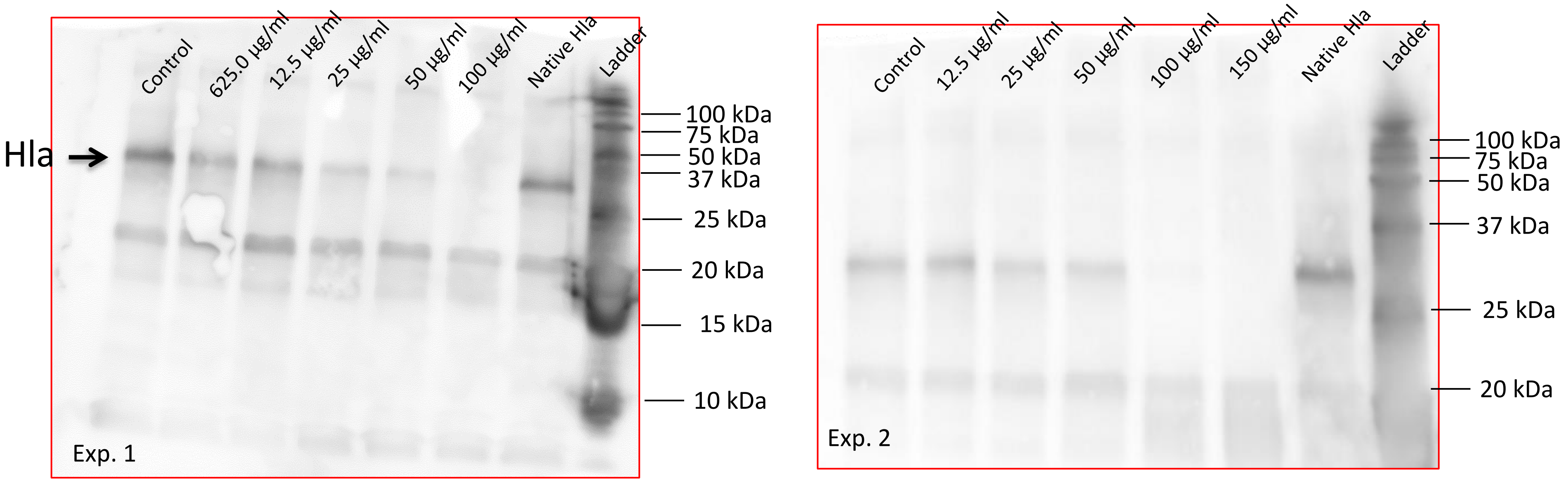

S1 Fig. Analysis of Hla expression at protein level in *S. aureus* USA300 treated with different concentration of TF3 for 24h. Experiment was done using Western blot method with anti-Hla antibody. A 45 µl/well of bacterial supernatant was separated on 8-16% gradient SDS-PAGE gels. Presented original WB images were acquired using the Azure cSeries digital system and auto-exposure settings.

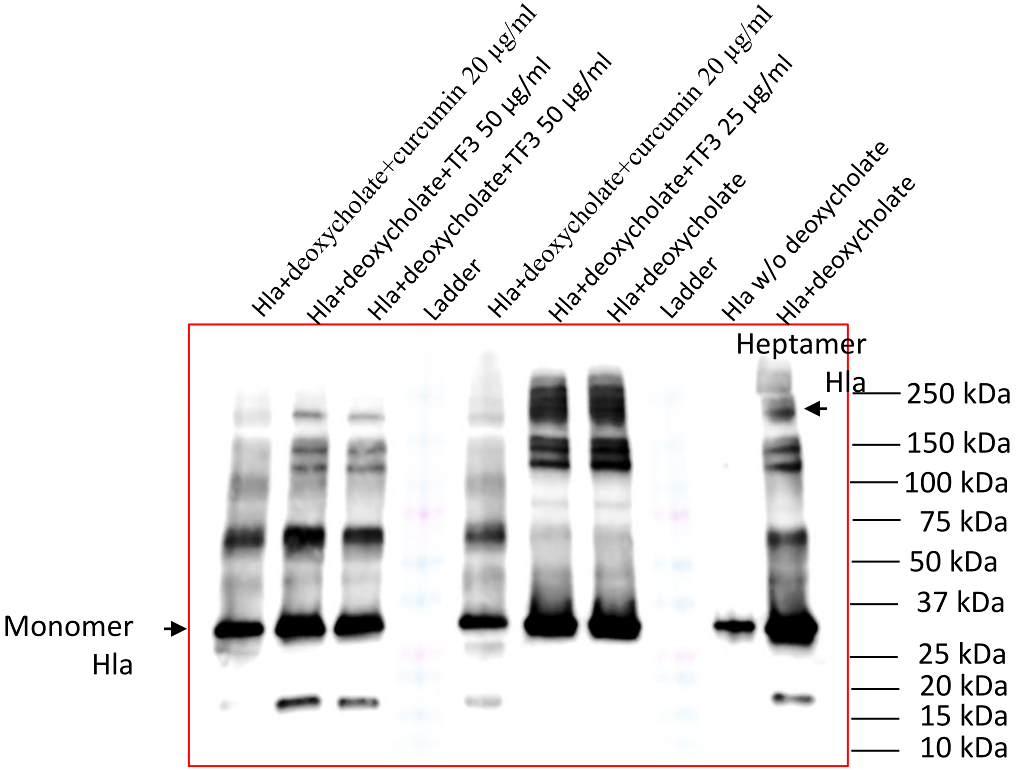

S2 Fig. Analysis of Hla oligomerization after treated with different concentrations of TF3. Experiment was done using Western blot method with anti-Hla antibody. A 35 µl/well of recombinant Hla was separated on 4-15% gradient SDS-PAGE gels as described in material and Method section. Presented original WB images were acquired using the Azure cSeries digital system and auto-exposure settings.

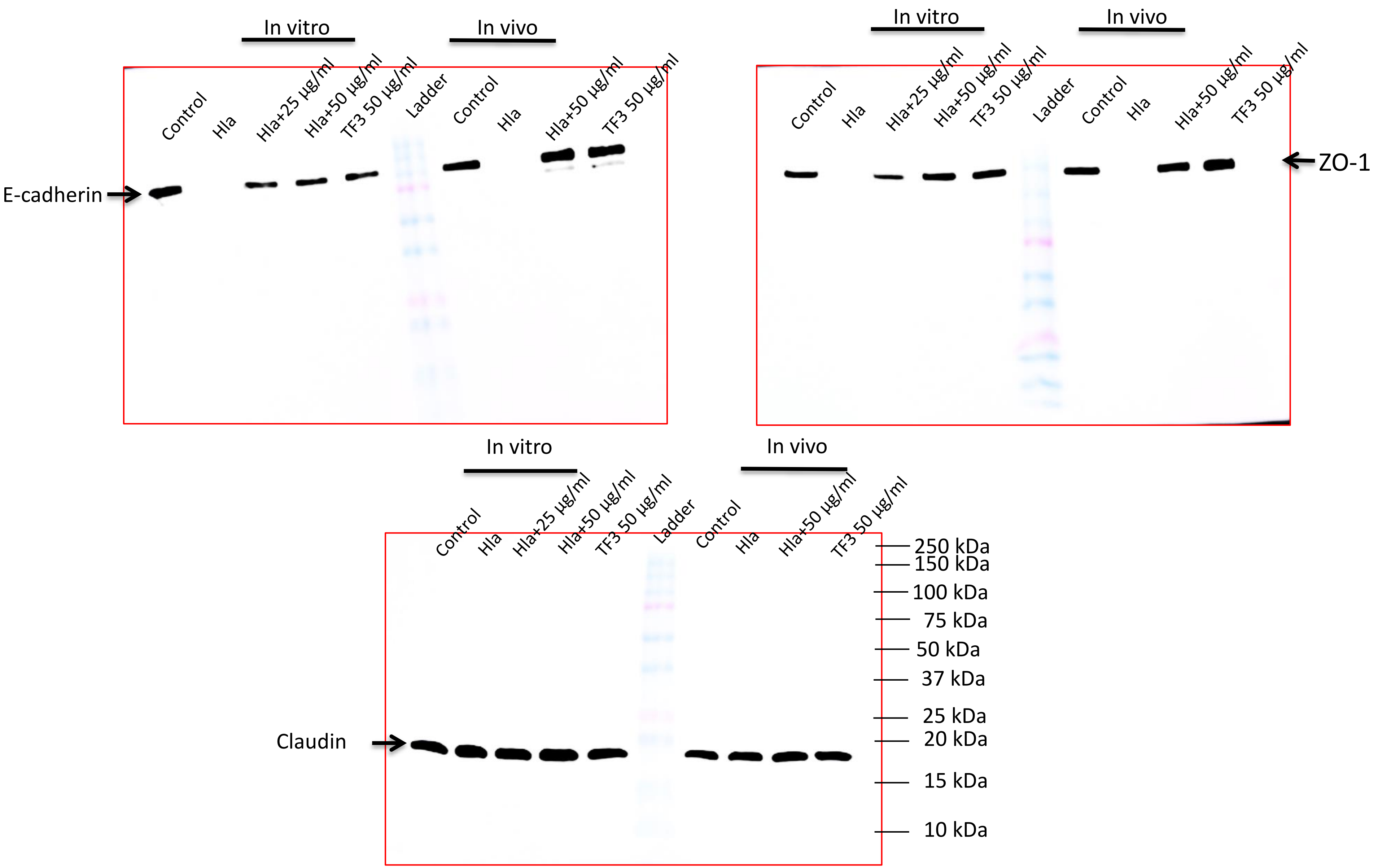

S3 Fig. Analysis of adherent and tight junctions molecules expression at protein level in human primary keratinocytes or mouse skin tissue treated with different concentration of TF3. Experiment was done using Western blot method with anti-E-cadherin, anti-ZO-1, and anti-claudin antibodies, which also served as a loading control. A 50 µg/well of protein was separated on 4-15% gradient SDS-PAGE gels. Presented original WB images were acquired using the Azure cSeries digital system and auto-exposure settings.
